# From Regional to National Clouds: TV Coverage in the Czech Republic

**DOI:** 10.1371/journal.pone.0165527

**Published:** 2016-11-08

**Authors:** Jan Sucháček, Petr Sed’a, Václav Friedrich, Renata Wachowiak-Smolíková, Mark P. Wachowiak

**Affiliations:** 1 Department of Regional and Environmental Economics, Faculty of Economics, VŠB—Technical University of Ostrava, Ostrava, Czech Republic; 2 Department of Mathematical Methods in Economics, Faculty of Economics, VŠB—Technical University of Ostrava, Ostrava, Czech Republic; 3 Department of Computer Science and Mathematics, Nipissing University, North Bay, Ontario Canada; Charles University, CZECH REPUBLIC

## Abstract

Media, and particularly TV media, have a great impact on the general public. In recent years, spatial patterns of information and the relevance of intangible geographies have become increasingly important. Gatekeeping plays a critical role in the selection of information that is transformed into media. Therefore, gatekeeping, through national media, also co-forms the generation of mental maps. In this paper, correspondence analysis (a statistical method) combined with cloud lines (a new visual analytics technique) is used to analyze how individual major regional events in one of the post-communist countries, the Czech Republic, penetrate into the media on a national scale. Although national news should minimize distortions about regions, this assumption has not been verified by our research. Impressions presented by the media of selected regions that were markedly influenced by one or several events in those regions demonstrate that gatekeepers, especially news reporters, functioned as a filter by selecting only a few specific, and in many cases, unusual events for dissemination.

## Introduction

The twenty-first century is quite legitimately known as the century of information. Informational needs are therefore relevant, and even comparable to physical needs. In spite of the growing importance of intangible geographies, spatial patterns of information have generally been underrated in the past [1]. Media play a relevant role in this context.

From the spatial point of view, media constitute a certain informational gate between “inner” groups, i.e. municipal and regional actors of territorial development, and “outer” groups, such as visitors, potential visitors, investors, non-regional entrepreneurs etc. [2]. Local and regional politicians, institutions, and entrepreneurial agents strive for media attention, and use media to address their voters, citizens or employees [3]. Conversely, these relevant actors involved in local and regional development obtain important information for their decision-making through media [4]. Moreover, agenda-setting or agenda-cutting is concomitant to contemporary media [5–12]. Not surprisingly, the media themselves became relevant actors of territorial development. Consequently, there is justification in talking about media-territorial development, rather than merely about territorial development [13].

Media can be distinguished as local, regional, and national (as well as, of course, international). Basically, there is a rank-territorial differentiation of media [14]. National media are obviously the most influential [15]. Regional news that resonates at the national level substantially co-determines the creation of mental maps. This spatial view on media is rather important.

The purpose of this paper is to analyze how individual major regional events, which fulfill certain attributes relevant to gatekeeping, penetrate into the media on a national scale by utilizing correspondence analysis (CA) and cloud lines, a new visual analytics technique. Correspondence analysis is a very effective tool in visualizing the relationship between two categorical variables, and is based on similar theoretical principles as exploratory factor analysis (EFA) [13]. Cloud lines provide an innovative interactive visualization and data exploration tool that, when used in conjunction with statistical methods, facilitates analysts’ insights and sense-making. In this paper, this visual technique is used to complement and to further develop results from CA. The research question is as follows: What is the character of regional events penetrating into media on the national scale? This question has not yet been analyzed in the literature.

The added value of this article consists in the application of correspondence analysis and cloud lines, which is novel in research dealing with spatial attributes of media. The data were acquired from the Czech Republic (population 10,436,560) [16], which is still in the post-transformation stage of its development. TV signals cover the whole country, and over two million people regularly watch the TV news reports analyzed in this study [17]. Furthermore, previous research has shown that the TV coverage agenda is to a large extent shared by the press and radio [18].

The authors geospatially analyze the media contributions (a contribution is a specific event, or a news story) related to the NUTS III (Nomenclature of Territorial Units for Statistics) self-governing regions in the Czech Republic. The thirteen (13) regions are: South Bohemia, South Moravia, Karlovy Vary, Hradec Kralove, Liberec, Moravian-Silesian, Olomouc, Pardubice, Plzen, Prague/Central Bohemia, Usti nad Labem, Vysocina, and Zlin. Regional and local identities are less intense in the Czech Republic, which is a typical characteristic of post-communist countries, and which is evident when comparing to countries with strong regionalism, such as Spain [19]. Analysis of political variables is beyond the scope of the present paper, as these variables have a different interpretation than in other Western countries.

It is worth noting that two NUTS III regions–Prague and Central Bohemia–were merged into one territory due to the better correspondence with natural geographical characteristics on the one hand, and suppression of the specific urban character of the capital city on the other. Unification of Prague and Central Bohemia into one territorial unit turned out to be correct and useful, as also found in previous research [20–21].

## Background

In recent years, a growing body of literature has dealt with the problem of media from the territorial point of view. Mass media influences spatial perception through journalistic cartography and spatial bias in news coverage. When certain news has an important geographic component, journalism is concerned with its spatio-temporal context. The spatial aspect of news is created by standard journalistic practice, which concerns national coverage, national interest, geographic stereotypes and accessibility to news events. As mass media provides live reporting from the scenes of news events, journalism requires spatial proximity, event proximity, and broadcast proximity [22–23].

From this perspective, the concept of gatekeeping is of utmost importance [24]. Gatekeeping, introduced by the social psychologist Kurt Lewin in 1943, is the process through which information is filtered. Gatekeeping is traditionally associated with communication, and has a variety of applications. For instance, in health care, information flows in hospitals are largely controlled by nurses in the operating room. Gatekeeping is consequently considered as a communication practice that has the potential to directly affect patient safety [25].

Gatekeeping examples range from a reporter deciding which sources should be included in a story to editors deciding which stories will be published. Gatekeeping also pertains to media owners, advertisers, and many other parties [26]. Also of importance is network gatekeeping theory, in which information dissemination and user behavior on the Internet is analyzed [27]. Berkowitz [28] observed that TV gatekeepers make decisions according to their “instincts”. He also concluded that “news judgements” and “news values” affect gatekeeping [29]. Bennett [30], for instance, utilized a modified approach to gatekeeping theory to argue that television news has shifted from hard to soft news for economic reasons. In his research, he pinpointed four news gates driven by the reporter, the news organization, its economics, and the news-gathering technology. It should be noted, however, that social networks are becoming increasingly relevant to this topic.

Gatekeeping is influenced primarily by the “value” of a topic. Galtung and Ruge [31] created a news values classification, which delimited news factors as follows:

- Frequency: An event that unfolds within a news medium’s publication cycle is more likely to be selected for publication than a one that takes place over a long period of time.
- Threshold: Events have to pass a certain qualitative “threshold” before being recorded at all; the greater the intensity (e.g. the more gruesome the murder, or the more casualties in an accident), the greater the impact, and the more likely it is to be selected.
- Unambiguity: The more clearly an event can be unequivocally understood and interpreted, the more likely it is to be selected.
- Meaningfulness: The culturally familiar is more likely to be selected.
- Consonance: Due to experience, the news selector may be able to predict which events will be newsworthy, thus forming a “pre-image” of an event, which in turn increases its chances of becoming news.
- Unexpectedness: Among meaningful events, the unexpected or rare event is more likely to be selected.
- Continuity: An event already in the news has a high likelihood of remaining in the news (even if its impact has been reduced) because it has become familiar and easier to interpret.
- Composition: An event may be included as news because it fits into the overall composition or balance of a newspaper or news broadcast, rather than exclusively because of its intrinsic newsworthiness.
- Reference to elite nations: The actions of elite or powerful nations are seen as more consequential than the actions of other nations.
- Reference to the élite: The actions of elite people, who are more likely to be famous, may be seen by news selectors as having more consequence than others, and, because of the former’s fame, news audiences may identify with them.
- Reference to persons: News that can be presented in terms of individual people, rather than abstractions, is likely to be selected.
- Reference to something negative: Negative events are generally unambiguous and newsworthy.

Although this list is far from complete, it still forms a benchmark *sui generis* in the field of media studies [32–34].

According to Graber [35], journalists rely on five criteria when choosing a news story:

- Strong impact: Local stories have a greater impact on the public than unfamiliar international events.
- Violence, conflict, disaster, or scandal is the second criterion: Topics such as murders, wars, shootings, or hurricanes capture the attention of the audience.
- Familiarity: News stories gain more attention if they have issues pertaining to the public or if they comprise familiar situations related to a large audience.
- Proximity: People pay closer attention to local news than they do to international or national events.
- Timely and novel: News should be something interesting that does not occur every day, or it should correspond to an event that is not a part of people's daily lives.

Media bias is the bias or perceived bias of journalists and news producers within mass media in the selection of events and stories that are reported and how they are covered. The term “media bias” implies a pervasive or widespread bias contravening the standards of journalism, rather than the perspective of an individual journalist or article. The direction and degree of media bias in various countries is widely disputed [36–38].

## Methods

The research focus in this paper is on the TV coverage represented by the evening news of three principal TV stations in the Czech Republic: Czech TV, TV Nova, and TV Prima. Czech TV is the public TV broadcaster, broadcasting six channels. TV Nova and TV Prima are private TV stations which began broadcasting in the 1990s. The empirical analysis is based on data purchased from Media Tenor, Ltd. which provides analytical services based on the analysis of content of media by evaluating qualitative and quantitative aspects of media contributions. Media data are statistically evaluated and analyzed with the Media Tenor Codebook. The company monitored stories from two Czech TV news outlets (“Události”, and “Události, komentáře” in Czech), TV Nova news (“Televizní noviny”), and TV Prima news (“Zprávy”) between 2004 and 2011.

In total, 54,667 contributions were monitored. Originally, there were thirty (30) thematic categories that were subsequently simplified into the final ten (10) categories, or pillars: Security, Social, Accidents, Economic, Public Affairs, Environmental, Justice, European and International, Education and Science, and Sport [13] (Table 1). A contribution can appear in only one category. As an event evolves over time, it can appear in different categories simply due to the development of news content (e.g. an event which was initially ranked under the Security pillar can later appear in the Justice pillar).

**Table 1:** Distribution of particular thematic pillars.

Pillars	Frequency	Percent
SECURITY	13,630	24.9
SOCIAL	12,826	23.5
ACCIDENTS	10,213	18.7
ECONOMIC	5,731	10.5
PUBLIC AFFAIRS	4,030	7.4
ENVIRONMENTAL	3,213	5.9
JUSTICE	2,147	3.9
EUROPEAN & INTERNATIONAL	1,106	2.0
EDUCATION & SCIENCE	983	1.8
SPORT	788	1.4
Total	54,667	100.0

Correspondence analysis and the cloud line visualization technique were used to support the gatekeeping concept. The research focus was mainly on individual events that attracted media attention. These techniques provide an intuitive interface for evaluating how individual major regional events that fulfill certain attributes relevant to gatekeeping penetrate into the media on a national scale.

### Correspondence Analysis

Correspondence analysis (CA) is a graphical pattern recognition method that allows the association relationships in the distribution of two categorical variables to be assessed and visualized. CA represents the relationships between categories of one or more variables in contingency tables, making it possible to describe the association of nominal and ordinal variables and to obtain a graphical representation of relationships in a multidimensional space [39]. The correspondence analysis diagram, i.e. correspondence or subjective map [40], shows the relationships between the rows and columns of a contingency table. Each row and each column is represented by one point in the map. The proximity of row points connotes the similarity of table rows, indicating rows that have a similar distribution profile. The same is true for column points. The mutual proximity of row and column points in the correspondence map shows combinations that appear more frequently than would be the case in an independent model. Beh [41] considered the greatest advantage of this method to be its ability to graphically illustrate the interconnectedness of the various categories. In the current study, the categorical variables which represent rows in contingency tables are regions or thematic pillars. Columns correspond to quarters of a specific year.

Rencher [42] emphasized that the basis for the creation of a subjective correspondence map are so-called latent variables. The location of points in a subjective map directly represents the association. The distance between points can be transferred onto the two-dimensional Euclidean plane, in which the points correspond to individual groups.

Greenacre [43] added that CA exhibits the correspondence of categories of individual variables, and provides a common view of row and column categories in the same dimensions. Unlike many other multivariate methods, CA allows the processing of categorized non-metric data, and even nonlinear relationships. Although it shares some commonality with factor analysis, the influence of various categories and their relative similarity or association with other categories of variables, instead of factors, can be visualized with CA [42].

According to Greenacre [43], the aim of CA is to reduce the multidimensional space of vectors of row and column profiles while maintaining the information contained in the original data. In a subjective mapping, two-dimensional (plane) or three-dimensional distances in Euclidean space are the most frequently used types of visualizations. Nevertheless, the Pearson's chi-square statistic is applied more frequently than Euclidean distances. Points close to rows indicate lines that have similar profiles in the entire line, and nearby column points indicate columns with similar profiles downwards through all rows.

Dispersion of points can be assessed according to indicators of inertia, which correspond to the weighted average of chi-square distances in row or column profiles [40]. These indicators represent the degree of variability between sections that are explained by a given dimension or given category. The difference of profiles, measured by the rate based on the chi-square statistics, is reflected in the chart as the distance between items of the same variable.

When creating a two-dimensional correspondence map, the information (variability) represented in the contingency table is reduced. The number of dimensions of the contingency table is equal to the smaller of the dimensions of contingency table minus one. The orthogonal axes (dimensions) of the correspondence map are therefore chosen so that this reduction is minimized. In other words, the correspondence map shows the maximum possible information. The first dimension always represents the largest part of the variability. Ideally, the both axes saturate at least 90% of total variability in data sample. In practice, it is considered to be sufficient at least 70% saturation [44].

In contrast to factor analysis, correspondence analysis does not usually determine the meaning of both dimensions (axes of correspondence maps). The purpose of correspondence analysis is not to identify factors (dimensions), but mainly to graphically describe and express the hidden inner association between rows and columns of a contingency table. This association allows generating a subjective correspondence map [40].

### Cloud Lines Visualization

Because of the size, complexity, and heterogeneity of time-series data encountered in many real-world problems, analysts frequently rely on interactive visualizations to gain insight [45]. Among the many time-series techniques, simple line graphs, small multiples, and horizon plots have been demonstrated to be useful [46–47]. However, many of the most actively-researched techniques assume continuous time-series, or data sampled from a continuous series. For time-based event and episodic interactive visualization, a new type of scatter plot, known as cloud lines [48], has been proposed to represent multiple time-series of large, dynamic, events in a small amount of screen space for easy analysis. This method is related to violin plots [49], and is intended to facilitate data analysis of event data while retaining the temporal dimension. Specifically, it aids in detecting important event episodes, exhibiting the structure of these event episodes with intuitive shapes, and allows interaction with the time series at a fine-grained level. With applications primarily in news publishing, network security, and financial services, cloud lines visualizations can be used in a variety of problems, including the ones presented in this paper. Cloud lines can also be adapted for continuous data, for example, in environmental monitoring time-series visualizations [50]. Additional modifications were made to the technique as originally presented in [48] for the purposes of the current study.

Cloud lines are essentially scatter plots where dependent variables are each shown as single lines, as a function of the independent variable, usually time. Events along the time axis–in this case the number of times a news item for a specific category was reported on a given day–are represented as circular markers that are colored according to their value, or whose radius and/or opacity reflects that value, resulting in lines of varying width and color. Cloud line plots for multiple properties or categories and regions can be shown simultaneously, and can be juxtaposed arbitrarily using a “drag and drop” repositioning mechanism.

For *N* days in the time series, let *x*_*i*_ denote the total number of events for day *i*, *i* = 1, …, *N* and let *r*(*x*) denote an importance function that maps the relative importance of an event or multiple events in a region or for a specific pillar to a numerical value:

$$r(x)=\frac{1}{Nh}\sum_{i=1}^{N}K\left(\frac{x-x_{i}}{h}\right).$$ (1)

Here, *h* denotes a selectable bandwidth *h*. The kernel *K*(·) is a non-negative zero-mean function that integrates to one. In the current work, the standard Gaussian kernel is used, although other kernels are possible. Therefore,

$$r(x)=\frac{1}{Nh}\sum_{i=1}^{N}\exp\left(-\frac{1}{2}{\left(\frac{x-x_{i}}{h}\right)}^{2}\right).$$ (2)

When *h* = 0, the original data are recovered with no kernel smoothing.

In Eq 1, *r*(*x*) denotes the importance function for the number of events over the time series, and *r*_*i*_ is the importance function for the number of events on day *i*. Each *r*_*i*_ can be mapped to the radius of the circle for the cloud line at day *i*, the opacity of the circle, or both. In the current study, each circle on the cloud line has constant opacity, with radius as the scaled value of *r*(*x*). Each category was assigned a unique color to allow the categories to be easily distinguished. To facilitate data exploration, a scaling factor is also provided. The scaled importance function *r*_*i*_′ is given as *r*_*i*_′ = *r*_*i*_^*α*^, where *r*_*i*_ denotes the scaled radius (0 ≤ *r*_*i*_ ≤ 1) at day *i*, and *α* ≥ 0 is a scaling factor. For small *α*, small radii are increased, thereby suppressing the range of radii. For large *α*, the range of radii is enhanced, allowing differences to be better distinguished.

**Fig 1:**
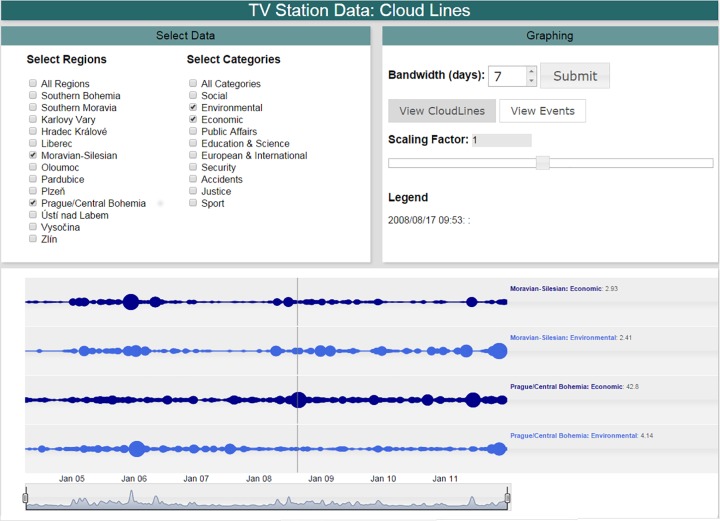
Cloud line visualization of two regions and two categories, grouped by region. The default bandwidth (*h* = 7 days) and scaling factor (*α* = 1) are used. Each category is colored uniquely. Data for the entire time duration are displayed.

**Fig 2:**
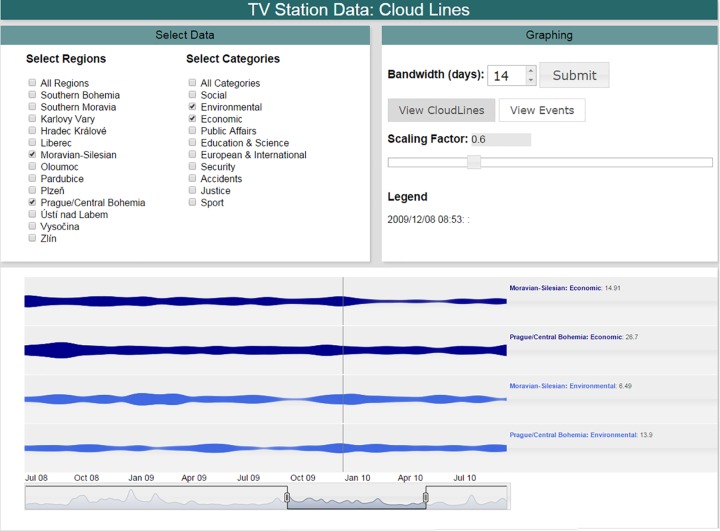
Cloud line visualization of two regions and two categories, grouped by category. A two-week bandwidth (*h* = 14 days) with scaling factor (*α* = 0.6) is used. The time selector is used to display zoomed data from June 18, 2008 to September 11, 2010.

The implementation described above is a highly interactive web-based system intended to facilitate collaboration amongst the researchers involved in the study. The system allows 14 × 11 = 154 different combinations of lines (13 regions, plus “all regions” [indicating national importance], and 10 categories, plus “all categories”). Regions and categories are divided into two panels on the graphical user interface (Fig 1). The bandwidth parameter is adjustable from 0 to 31 days (maximum number of days in a month), with a default of one week (7 days). Analysts have the ability to juxtapose cloud lines in any order by repositioning (“dragging and dropping”) the individual cloud lines on the window display. A time-selection control below the cloud lines can be used to zoom into any time period represented in the data (Fig 2). Scaling factors range from 0.1 to 2. A graphical comparison of the effect of different bandwidth and scaling parameters is shown in Figs 1 and 2. The system is primarily written in JavaScript. The open source Dygraphs JavaScript library (www.dygraphs.com) was custom-modified to support cloud lines. For performance, kernel density estimation is performed in PHP on the server computer.

## Results

The analysis in this section focuses on several events that had been resonating in the media between 2004 and 2011. We compared a “footprint” of these events on an analyzed region chart and on a nationwide chart.

### Testing the Dependency of Distribution of News between Thematic Pillars and Regions over Time

In order to examine the differences in the development of events among NUTS III regions, or among thematic pillars, it is necessary to apply a statistical test of independence. This test verifies whether frequency distribution of news in TV media varies in time, among regions, and among thematic pillars.

We examined the following hypotheses:

*H*_1_: Frequency distribution of news in the TV media differs among different regions over time.
*H*_2_: Frequency distribution of news in the TV media differs among different thematic pillars over time.

These two hypotheses are considered to be alternatives from the perspective of the Neyman-Pearson methodology of statistical tests. The corresponding null hypothesis would substitute the words “does not differ among” for “differs among”. The differences among regions are inherent attributes of the space. This applies to both the material and intangible perspectives [20–21, 51].

As a first example, we studied a well-known incident concerning the Faculty of Law at the University of West Bohemia in Pilsen (“Plzeň” in Czech). This incident was a media-exacerbated scandal with “turbo students” (a phenomenon in which students accelerate their course of study to complete degree requirements in extraordinarily short times) that involved senior politicians in the Czech Republic [52]. The students obtained their degrees after only a few months of study.

With regard to this affair that culminated in 2009, statistical tests were confined to particular quarters of that year only. Overall, 9,299 contributions were monitored in 2009 from a total of 54,667 contributions recorded in 2004–2011. Even if (0%) the timeframe were extended to the entire period of the research, i.e. 2004–2011, the test results would not have changed.

In both cases, the asymptotic chi-squared test of independence in the contingency table was applied. Moreover, the symmetrical Cramer’s V statistic was applied as a measure of the intensity of dependence in case of rejection of the null hypothesis (i.e. the adoption of hypotheses *H*_1_ and *H*_2_). All tests were performed at 5% significance level.

Table 2 shows the chi-squared test results corresponding to hypothesis *H*_1_. All necessary conditions for performing the asymptotic chi-squared test were met (Table 2, note a). The number of cells in contingency table was 52 since we analyzed the frequency distribution among thirteen NUTS III regions and four quarters of 2009. The asymptotic significance level of the test reached 1.31×10^−27^, so that the null hypothesis of independence can be rejected at 5% level of significance. Therefore, the hypothesis *H*_1_ cannot be eliminated. The value of the Cramer´s coefficient V = 0.088 shows that the observed dependence is very weak [53].

**Table 2:** Chi-Squared test corresponding to hypothesis *H*_1_.

	Value	df	Asymp. Sig. (2-sided)
Pearson Chi-Square	216.374^a^	36	1.31 × 10^−27^
Cramer’s V	0.088		
N of valid cases (contributions in 2009)	9299		

a. 0 cells of 52 (0%) have expected count less than 5. The minimum expected count is 74.18.

It can be concluded that the frequency distribution of news in the TV media differs among different regions over time. Nevertheless, the observed dependence is very weak. Despite the similarity of development of the number of news over time, it is important to examine and to compare the number of contributions based on particular regions in relation to time.

Table 3 shows the chi-squared test results corresponding to hypothesis *H*_2_. All necessary conditions for carrying out the asymptotic chi-squared test were met (Table 3, note a). The number of cells in contingency table was 40. The frequency distribution among ten pillars and four quarters of 2009 was analyzed. Since the asymptotic significance level of the test reached 2.25×10^−63^, the null hypothesis of independence can be again rejected at 5% significance level. Thus, the hypothesis *H*_2_ cannot be eliminated either. The value of the Cramer´s coefficient V = 0.116 shows that the observed dependence is significant, but again very weak [53].

**Table 3:** Chi-Squared test corresponding to hypothesis *H*_2_.

	Value	df	Asymp. Sig. (2-sided)
Pearson Chi-Square	377.108^a^	27	2.25 × 10^−63^
Cramer’s V	0.116		
N of valid cases (contributions in 2009)	9299		

a. 0 cells of 40 (0%) have expected count less than 5. The minimum expected count is 26.82.

It can also be concluded that the frequency distribution of contributions in TV media differs among different thematic pillars over time, even if the observed dependence is weak. Nonetheless, it is still important to examine and to compare the development of the number of posts over time depending on the thematic pillars.

Conclusions drawn from both statistical tests enable us to examine the differences in the development of the relative number of posts in the nationwide TV programs across regions or thematic pillars. These differences can be visualized by descriptive tools such as correspondence analysis complemented with cloud lines.

### Application of Correspondence Analysis to Search for Information Clusters

If we appropriately use categorized time in terms of categorical variables (months or quarters, for example), correspondence analysis allows us to detect clusters of events or news in a time series that occur more frequently than others (at other times, for other regions, or for other thematic pillars). For the purpose of the correspondence analysis, we utilized the data sample containing 54,667 contributions described in Table 1.

**Fig 3:**
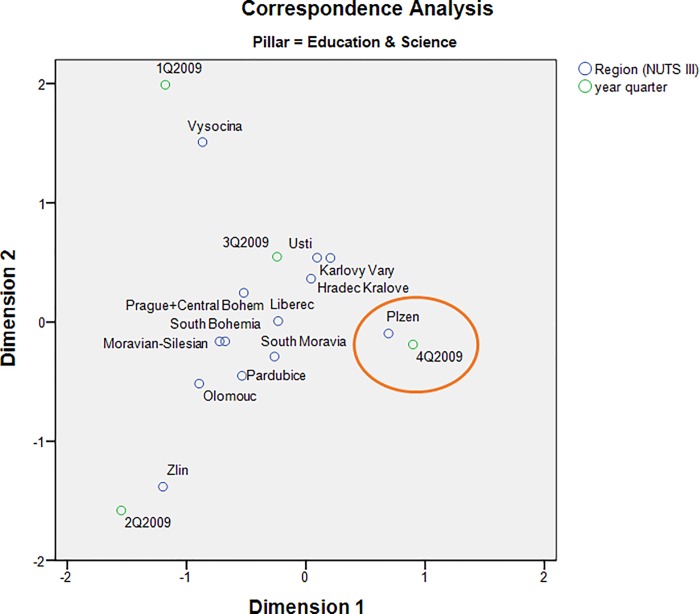
Correspondence map showing all NUTS III regions within the Education & Science pillar.

The correspondence map showing all NUTS III regions within the Education & Science pillar (Fig 3) demonstrates the proximity of the Pilsen region and the 4th quarter of 2009. This fact corresponds to the period when the affair of the Faculty of Law of the University of West Bohemia in Pilsen was greatly publicized (as will be demonstrated below through cloud lines visualization).

**Fig 4:**
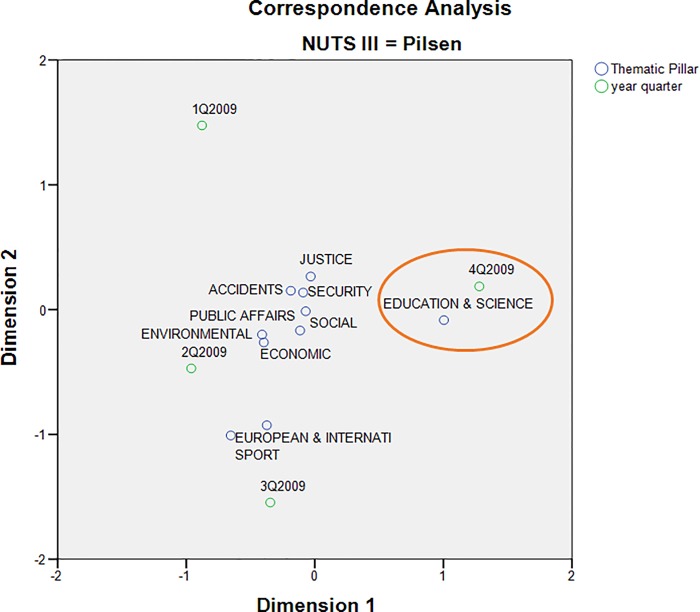
Correspondence map showing all pillars within Pilsen region.

The correspondence map showing all thematic pillars for Pilsen region presents a different view of the same event. While sports news dominated in the 3rd quarter, reflecting the fact that Pilsen hosted several international sporting events in the summer of that year, the Education & Science pillar clearly prevailed again in the 4th quarter (Fig 4).

**Fig 5:**
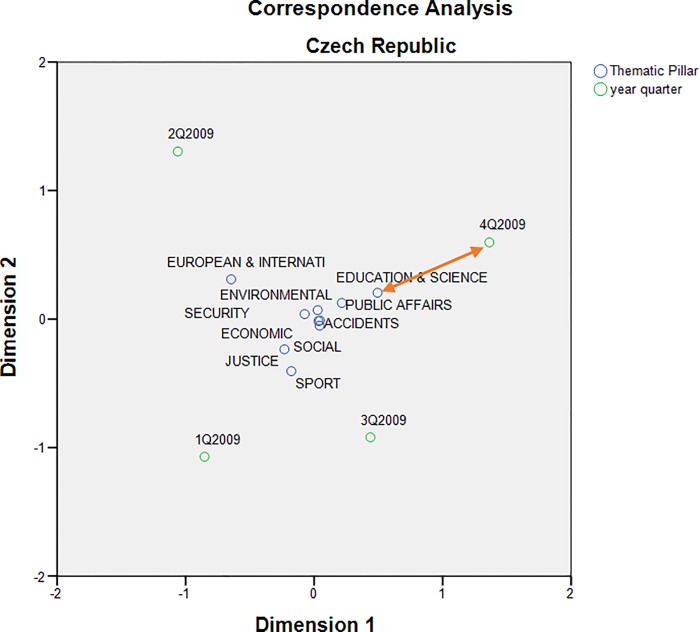
Correspondence map showing all pillars within the Czech Republic.

When extending Fig 4 for the entire Czech Republic, it can be seen that the thematic pillars form a more homogeneous group (Fig 5). However, it is obvious that the fourth quarter of 2009 is closest to the Education & Science pillar. This fact suggests that the affair of the Faculty of Law at the University of West Bohemia has exceeded the regional framework of the Pilsen region.

The quality of displayed data in correspondence maps may be characterized by the cumulative saturation of data variability in both dimensions. Axis 1 and Axis 2 in Fig 3 explain 83.5% of the total variability in the corresponding contingency table. Similarly, both axes in Fig 4 represent 98.5% of variability. Further, 71.1% of the data variability is explained by both axes in Fig 5. The quality of all three correspondent maps seems to be relatively high [44].

### Correspondence Analysis and Cloud Lines

The distances in the correspondence map are not genuine linear measures of the intensity of the relationship between displayed items. However, cloud lines allow visualization of the real density or intensity of events in particular NUTS III regions and thematic pillars, even in a broader timeframe. In addition, cloud lines treat time as a real continuous variable, with resolutions in units of days. However, for the purpose of CA, it is necessary to categorize time into larger units of time, i.e. months or quarters. Therefore, cloud lines visualizations complement CA by providing finer grained time resolution. In that way, we can observe whether an analyzed event arises in the context of the other news from the given region, or if the same event is subdued by other news from other territories. Such an observation can be expressed graphically using cloud lines in the form of a “bubble” or a “cloud” on the regional chart.

**Fig 6:**
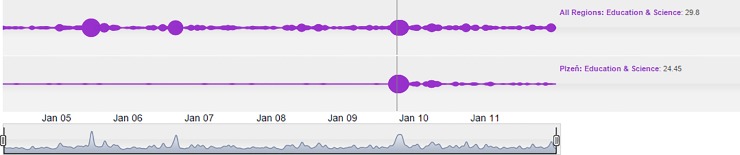
Cloud lines visualization showing penetration of a regional (Pilsen/Plzeň) story into the national media for the Education & Science pillar.

We used cloud lines to visualize several news events. For instance, concerning the incident at the Faculty of Law at the University of West Bohemia in Pilsen studied above, this affair erupted in the Fall of 2009, and has been lingering in the media until 2011, as demonstrated in Fig 6.

Fig 6 shows that no significant news events in the Education & Science category for 2009 can be observed before October 2009 in the Pilsen region. Afterwards, the abovementioned scandal occurred–demonstrated by a large “bubble”–whose effects linger for some time. It should be stressed that the penetration of the event on the national scale is clearly visible.

This event is concomitant to a post-transformation country, the Czech Republic, with institutions that are not yet mature. Moreover, the nature of this event is largely in compliance with the gatekeeping concept. A cluster of events of similar significance could already be observed starting in 2005 in all regions. However, this was the result of increased news interest in the Education area in virtually all regions. The causes of the events were the acts of the Ministry of Education, especially its endeavor to repeal a compulsory state exam in mathematics in high schools. Those activities by the Minister of Education Petra Buzková triggered a controversial media response.

A similar media scandal in the area of sports is related to the FIS Nordic World Ski Championship held in the Liberec region in 2009. This sporting event is associated with Kateřina Neumannová, the Olympic Champion and World Champion in cross-country skiing. She was appointed as the chairman of the organizing committee, and using that power, she suspended the existing organizing team in order to promote her own associates into the organizing committee. The championship subsequently faced a lack of spectator interest, which resulted in a severe economic loss. This happened in spite of the fact that the organizers promised full grandstands and a substantial economic benefit. A dispute in the media between the organizers and their opponents, who were former members of the organizing committee, occurred immediately after the championship. Some criminal charges were also laid.

**Fig 7:**
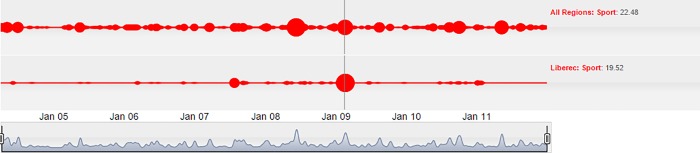
Cloud lines visualization showing a regional issue in the Sports pillar, which was reflected more strongly nationally than locally.

The first major “cloud” in the Liberec region, seen in 2007, is associated with publishing the information that Kateřina Neumannová would participate in the organizing committee of the World Ski Championship. This news had no effect at the national scale. However, the economic problems related to the championship became apparent in 2009, as shown in Fig 7. The cloud “bubble” appearing at the national scale in 2008, which is even stronger than the Neumannová affair and the World Ski Championship in Liberec, may be associated with the Olympic Games in Beijing and the European Championship in soccer. Those events can be considered as the two major international sporting events, but they were not reflected in the regional media.

**Fig 8:**
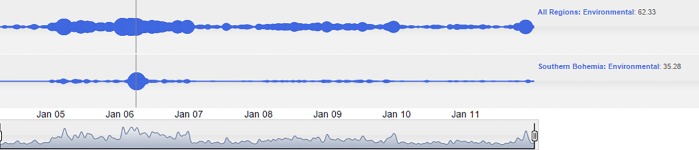
Penetration of the Temelin nuclear power plant affair into national news.

The affair of the Temelin nuclear power plant in the South Bohemia region can serve as an additional example of penetration of regional issues into the media at the national level. Although environmental issues are quite often reported in the media, the cloud lines visualization on the national scale seem relatively complex. In 2006, a clear intrusion of the regional issues–in this case from South Bohemia–into the national level can be observed. The Temelin nuclear power plant was officially launched in 2006. This event was accompanied by numerous problems before the plant was launched. In addition, there were many protests in neighboring Austria, and even a blockade of the national borders between the Czech Republic and Austria, which is demonstrated by the cloud lines in Fig 8.

**Fig 9:**
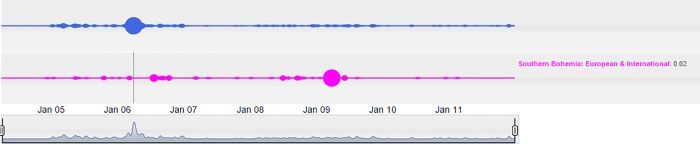
Visualization showing that the Temelin affair was assessed as an environmental issue, given the relative lack of activity in the European & International pillar.

The bottom cloud line chart shows that the uproar regarding the Temelin nuclear power plant lasted almost the entirety 2006. It is also of interest to compare the Environmental and the International pillars in connection to Temelin. It turned out that the Temelin affair was assessed as an environmental issue. However, there was a period of a relative calm in the area of international news at the same time, as seen in Fig 9.

**Fig 10:**
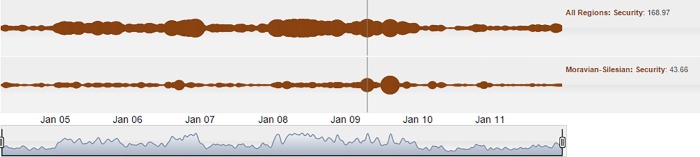
Cloud line visualization of a long-lived Moravian-Silesian arson incident in the Security pillar.

The last example is related to an attack in the Moravian-Silesian region that occurred in April 2009. Four young men set fire to a house where a large family was sleeping. A two-year-old girl suffered serious burns. This act was spontaneously condemned by most of the Czech population, and has been debated in the media for a long period of time, as seen in Fig 10.

This event is less obvious in the context of other criminal acts on a national scale than in its own region. Cloud lines of the Security pillar have high volatility for all regions. However, its footprint is also evident on a national scale. The period of about four months later (August 2009), when the perpetrators were accused of a racially motivated crime and remanded into custody, has an even more visible track.

**Fig 11:**
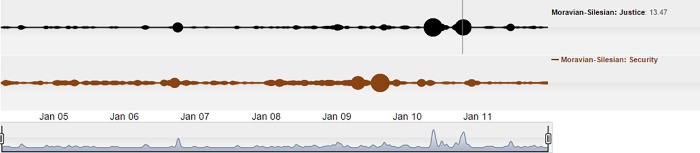
Penetration of the Moravian-Silesian arson event from the Security pillar to the Justice pillar from the arrest of the perpetrators to the subsequent juridical process.

Afterwards, the whole issue spilled over into the Justice pillar. Two cloud bubbles in this pillar coincide with the start of the juridical process in May 2010 and the delivery of the judgment in October 2010, as demonstrated in Fig 11.

In summary, it can be observed that several regional TV news stories that appeared on national TV broadcasting attracted wide attention. What they have in common is their specific character, which is attractive from the perspective of gatekeeping. Moreover, one or several single events usually affect media portrayals of regions at the national level. In light of the research question, our expectations were confirmed.

## Discussion

Extraordinary and unusual news, generally negative in character, turned out to be symptomatic for regional coverage appearing within national TV broadcasting in the Czech Republic. We are even justified in speaking of the tabloid character of news concerning localities and regions. Media portrayals of individual territories are gradually losing their unique, regional character, which would be consonant with the varieties of activities and life in these territories. It is also reasonable to assume a distinct lack of news oriented to genuine local and regional development. Moreover, one or more single events usually tend to affect media portrayals of whole territories.

These findings can be accounted for by the gatekeeping process, which represents a certain benchmark in the field of media studies. It takes into consideration the limited amount of time and space available in any medium for its daily news presentations to its audience. That is the primary reason that only selected news reports appear on TV screens, which in turn shape and often negatively skew media portrayals of localities and regions. Persons involved in media are acting as gatekeepers between true events and media messages to the general public. Our results are in agreement with Graber [35], in partial compliance with Galtung and Ruge [31], but much less so with Bennett [30].

This paper demonstrated that neither hypothesis *H*_1_ nor *H*_2_ can be eliminated. This means that we cannot reject the idea that the frequency distribution of news in the Czech TV media differs among different regions over time. The same applies to the frequency distribution of news in the Czech TV media, which differs among different thematic pillars over time [20–21].

For example, correspondence analysis captures the relation between news reports in the media and thematic areas, and answers questions such as whether these reports will focus more on societal problems than on tabloid scandals. It can also show which news reports are thematically similar and in which ways they differ [13].

Using correspondence analysis plots between the same variables at different times, the dynamics of these relationships can be captured. For example, which variables retain their main focus points and which evolve over time (and in what direction) can be shown. Those plots can be consulted for a time instant when, as seen through cloud lines, there is relative media calm, and when there is some crucial affair or important issue (e.g. unsuccessful hockey players at the World Championships, upcoming elections, etc.).

This paper is original not only in its descriptive analysis of the Czech Republic, but also because it presents a thorough discussion of what this analysis adds to the scientific debate about gatekeeping. Based on the data from the Czech Republic, we clearly observed the importance of the spatial dimension of gatekeeping, in that regional events manifested themselves significantly on the national scale. This aspect has not been reported in the previous literature on gatekeeping. A further innovative aspect of this research consists in the combined application of cloud lines and correspondence analysis in the sphere of TV coverage within a spatial context. Both methods enhance the study of media events via intuitive visual presentation.

## Conclusion

The purpose of this study was to confirm the growing relevance of intangible geographies. Media can be perceived as rather significant and at the same time, an increasingly aggressive institution. Our research focused on the Czech Republic, a small post-transformation country in Central Europe, for which TV broadcasting is still of enormous importance, as the country’s inhabitants are less sophisticated in terms of media. The conception of gatekeeping turned out to be of primary importance. Our research revealed that events on regional level became significant also on the national scale. Media data can be studied from different perspectives by complementary quantitative and qualitative methods. While cloud lines show dynamic phenomena, correspondence analysis examines and displays the relationships between qualitative variables at a particular time instant. Correspondence maps and cloud lines visualizations used in this paper are part of a visual analytics approach: interactive visualizations can be combined with statistical techniques to gain new insights. Such an approach can potentially be used on any media data from any country, and has a very wide spectrum of applications in broadcasting.
